# Risk factors for deep vein thrombosis even using low-molecular-weight heparin after total knee arthroplasty

**DOI:** 10.1186/s43019-021-00109-z

**Published:** 2021-09-07

**Authors:** Joon Kyu Lee, Kee Byoung Lee, Joong Il Kim, Gun Tae Park, Young Chang Cho

**Affiliations:** 1 Department of Orthopaedic Surgery, Konkuk University Medical Center, 120-1, Neungdong-ro, Gwangjin-gu Seoul, 05030 South Korea; 2 Research Institute of Medical Science, Konkuk University School of Medicine, 120-1, Neungdong-ro, Gwangjin-gu Seoul, 05030 South Korea; 3 Department of Orthopaedic Surgery, Cheongju St. Mary’s Hospital, 173-19 Jusung-ro, Cheongwon-gu, Cheongju-si, Chungcheongbuk-do 28323 South Korea; 4 Department of Orthopaedic Surgery, Hallym University Kangnam Sacred Heart Hospital, 1, Singil-ro, Yeongdeungpo-gu, Seoul, 07741 South Korea; 5 Department of Orthopaedic Surgery, Hallym University Sacred Heart Hospital, 22 Gwanpyeong-ro, 170beon-gil, Dongan-gu, Anyang-si, Gyeonggi-do 14068 South Korea

**Keywords:** Deep vein thrombosis, Total knee arthroplasty, Anticoagulant agent, Low-molecular-weight-heparin, Risk factor

## Abstract

**Background:**

With an increase in deep vein thrombosis (DVT) following total knee arthroplasty (TKA) in the Asian population, most surgeons today use a form of prophylactic anticoagulant agents in patients after TKA. Nevertheless, DVT occasionally develops even in these patients with prophylaxis. The purpose of this study was to identify the risk factors for DVT after TKA in cases of postoperative low-molecular-weight heparin (LMWH) use.

**Methods:**

We designed a retrospective study with 103 patients who underwent primary TKA. From the second postoperative day, 60 mg of LMWH was subcutaneously injected into the patients daily. On the seventh postoperative day, patients had computed tomography angiography to check whether they had DVT. Regarding risk factors, we investigated patients’ gender, age, surgical site (unilateral/bilateral), body mass index, method of anesthesia, preoperative hypertension, diabetes, hypercholesterolemia status, and prothrombin time/international normalized ratio from electronic medical records. We analyzed the statistical significance of these risk factors.

**Results:**

Statistically significant factors in the single-variable analysis were surgical site (unilateral/bilateral), body mass index, preoperative hypertension status, and anesthesia method. Multiple logistic regression analysis with these factors revealed that the surgical site (unilateral/bilateral, *p* = 0.024) and anesthesia method (*p* = 0.039) were significant factors for the occurrence of postoperative DVT after TKA.

**Conclusions:**

Patients undergoing simultaneous bilateral TKAs and patients undergoing TKA with general anesthesia need more attention regarding DVT even with chemoprophylaxis using LMWH after TKA.

## Introduction

Deep vein thrombosis (DVT) is one of the typical complications that can occur after total knee arthroplasty (TKA) [1, 2]. The management of DVT has long been widely debated. While some insist that calf DVT only needs to be closely monitored, others support some form of treatment to prevent proximal transmission, which may even lead to life-threatening pulmonary embolism (PE) [3–7]. Numerous attempts have been made to prevent these complications, and various studies have been conducted on this topic [3, 8–10]. Usually, anticoagulant agents are used with or without other methods to prevent DVT. However, quite a few surgeons have been reluctant to use these agents because of bleeding, swelling, delayed healing of the incision site, and increased risk of infection [11, 12].

For Asians, there have been several studies that reported a reduced rate of DVT after TKA compared to the Western population [4, 5, 13–18]. Therefore, there were debates on whether prophylactic treatment for DVT is actually needed after TKA for Asians [6, 19–21]. However, studies have shown that the DVT rate after TKA in the Asian population is increasing, with one study reporting a DVT incidence of up to 62.5% without prophylaxis [22–24]. Because serious and fatal results can happen if DVT is not treated properly [2], it is now widely accepted that some forms of DVT prevention should be performed, even though some forms of DVT prevention is not up to Western countries [21].

Although these anticoagulant agents certainly reduce DVT occurrence to some extent, there are occasions of DVT occurrence even with the usage of these agents [6]. There are several factors considered to be critical to the occurrence of DVT even with the prophylaxis, but there is a lack of literature reporting factor analysis for DVT in this situation, especially in the Asian population. Chung et al. reported DVT incidence after TKA using postoperative factor Xa inhibitor in Koreans, but they also acknowledged insufficient data in Asians regarding the DVT incidence with chemoprophylaxis [6].

The purpose of this study was to identify the risk factors for DVT after TKA in cases of postoperative chemoprophylaxis.

## Materials and methods

### Study subjects

Patients who underwent primary TKA between March 2014 and June 2017 for degenerative osteoarthritis were subjects of this study. Patients taking antiplatelet agents or anticoagulant drugs more potent than aspirin because of their underlying cerebrovascular disease or heart disease were excluded from the study. Patients on low-dose aspirin were guided to stop taking aspirin seven days before the operation. Patients receiving second-stage TKAs were also excluded regardless of previous postoperative DVT status. A total of 103 patients were included in this study.

### Surgical information

A single surgeon carried out all the surgeries on the subjects. An identical TKA procedure was performed in all cases. A midline skin incision, paramedial arthrotomy was performed. Only posterior-stabilized implants with a fixed bearing design were used. All implants were fixed with a cementing technique. Patella resurfacing was not performed. No navigation devices or patient-specific instruments were used. Tranexamic acid was not used at any time prior to, during, or after surgery.

### Chemoprophylaxis

We used low-molecular-weight heparin (LMWH, enoxaparin sodium, Clexane inj. 60 mg/0.6 ml, Sanofi-Aventis Korea) after TKA for the prevention of DVT along with some mechanical devices [25]. After the operation, we used 60 mg of LMWH daily by injecting it into the subcutaneous layer after removal of the drain, which took place on the second postoperative day. Stockings and pneumatic mechanical pumps were used immediately after the operation.

### Rehabilitation

Range-of-motion (ROM) exercise of the operated knee and walker-assisted ambulation were begun on the second postoperative day. From the fourth postoperative day, routine postoperative rehabilitation treatment, such as gentle passive ROM exercise, parallel-bar gait training, and quadriceps-strengthening exercise, was given daily in the physical therapy room. The rehabilitation protocol was identical for both unilateral and bilateral cases.

### Evaluation

Every patient had lower leg computed tomography (CT) angiography on the seventh postoperative day for the evaluation of DVT. One experienced radiologist confirmed the DVT diagnoses. DVT was defined as a thrombus either in the femoral, superficial femoral, popliteal vein, or veins distal to the popliteal vein. Regarding risk factors for DVT, gender, age (divided into three groups: 64 years and 364 days, 65 years and 0 days to 74 years and 364 days, 75 years and 0 days and older), surgical site (unilateral/bilateral), body mass index (BMI, divided into three groups: under 25 kg/m^2^, 25–30 kg/m^2^, and 30 kg/m^2^ or more), type of anesthesia, the preoperative status of hypertension, diabetes, and hypercholesterolemia, and prothrombin time/international normalized ratio (PT INR, divided into four groups: under 0.95, 0.95–1.00, 1.00–1.05, 1.05 or more) were checked from the electronic medical records. The factors were selected through a literature review.

### Statistical analysis

Single-variable analyses with the chi-square test or Fisher’s exact test were performed to evaluate the relationship of each factor to DVT. Then, with the factors that had significant relationships (*p* < 0.2), multiple logistic regression analyses were done to determine the factors that had a significant impact on the occurrence of DVT after TKA [26–28]. Analyses of unilateral TKA cases were additionally performed. A *p*-value of < 0.05 was considered significant.

The ethics committee of the Hallym University Sacred Heart Hospital approved this study (2017-I140). Written informed consent was exempted by the Institutional Review Board (IRB).

## Results

Among the 103 patients enrolled in this study, 22 patients had positive DVT results. Sixteen patients had DVT only at the veins distal to the popliteal vein. There was one patient each with DVT only at the femoral vein and DVT only at the superficial femoral vein. Two patients had DVT at the popliteal vein alone, and two patients had DVT at both the popliteal vein and the vein distal to the popliteal vein.

In the single-variable analyses, surgical site (unilateral/bilateral) (*p* = 0.005), BMI (*p* = 0.043), preoperative hypertension (*p* = 0.078), and type of anesthesia (*p* = 0.006) were the meaningful factors (Table 1). A multiple logistic regression analysis with these factors revealed that the unilateral/bilateral surgical site (*p* = 0.024) and type of anesthesia (*p* = 0.039) were significant factors for the occurrence of postoperative DVT after TKA (Table 2).

**Table 1:** Single-variable analysis results of factors for the development of deep vein thrombosis

Factor		Positive	Negative	*p* value
Deep vein thrombosis		22	81	
Gender	Male	4	8	0.279^*^
	Female	18	73	
Age (years)	< 65	3	21	0.773^**^
	≥ 65 to < 75	16	35	
	≥ 75	3	25	
Unilateral/bilateral	Unilateral	15	74	0.005^**a^
	Bilateral	7	7	
Body mass index (kg/m^2^)	< 25	5	38	0.043^**a^
	≥ 25 to < 30	13	35	
	≥ 30	4	8	
Type of anesthesia	Epidural	3	37	0.006^*a^
	General	19	44	
Hypertension	+	18	48	0.078^*a^
	–	4	33	
Diabetes	+	3	20	0.389^*^
	–	19	61	
Hypercholesterolemia	+	4	9	0.468^*^
	–	18	72	
Prothrombin time/international normalized ratio	< 0.95	8	29	0.554^**^
	≥ 0.95 to < 1.00	8	22	
	≥ 1.00 to < 1.05	4	19	
	≥ 1.05	2	11	

^*^Fisher’s exact test

^**^Pearson’s chi-square test

^a^Significant relationship in single-variable analysis (*p* < 0.2)

**Table 2:** Multiple logistic regression analysis results of factors for the development of deep vein thrombosis.

Factor	Odds ratio	95% confidence interval	*p* value
Unilateral/bilateral	4.751	1.23218.320	0.024^a^
Hypertension	2.591	0.7389.098	0.137
Type of anesthesia	4.210	1.07516.482	0.039^a^
Body mass index (kg/m^2^)			0.142
≥ 25	(Comparator)		
≥ 25 to < 30	3.395	0.94412.211	0.061
≥ 30	3.600	0.69118.756	0.128

^a^Significant factor in multiple logistic regression analysis (*p* < 0.05)

In analyses including only the unilateral cases, gender (*p* = 0.085), BMI (*p* = 0.021), preoperative hypertension (*p* = 0.156), and type of anesthesia (*p* = 0.084) were similarly the meaningful factors in the single-variable analyses (Table 3). However, a multiple logistic regression analysis with these factors revealed no truly significant factor for the occurrence of postoperative DVT (Table 4).

**Table 3:** Single-variable analysis results of factors for the development of deep vein thrombosis in unilateral cases

Factor		Positive	Negative	*p* value
Deep vein thrombosis		15	74	
Gender	Male	4	7	0.085^*a^
	Female	11	67	
Age (years)	< 65	2	19	0.592^**^
	≥ 65 to < 75	12	33	
	≥ 75	1	22	
Body mass index (kg/m^2^)	< 25	3	33	0.021^**a^
	≥ 25 to < 30	8	35	
	≥ 30	4	6	
Type of anesthesia	Epidural	3	35	0.084^*a^
	General	12	39	
Hypertension	+	12	44	0.156^*a^
	–	3	30	
Diabetes	+	3	19	0.754^*^
	–	12	55	
Hypercholesterolemia	+	3	7	0.363^*^
	–	12	67	
Prothrombin time/international normalized ratio	< 0.95	4	27	0.877^**^
	≥ 0.95 to < 1.00	5	20	
	≥ 1.00 to < 1.05	4	16	
	≥ 1.05	2	11	

^*^Fisher’s exact test

^**^Pearson’s chi-square test

^a^Significant relationship in single-variable analysis (*p* < 0.2)

**Table 4:** Multiple logistic regression analysis results of factors for the development of deep vein thrombosis in unilateral cases

Factor	Odds ratio	95% confidence interval	*p* value
Gender	3.646	0.76717.329	0.104
Hypertension	2.374	0.55210.202	0.245
Type of anesthesia	2.319	0.5449.881	0.255
Body mass index (kg/m^2^)			0.094
≥ 25	(Comparator)		
≥ 25 to < 30	2.333	0.52910.284	0.263
≥ 30	8.057	1.22852.863	0.030

significant factor in multiple logistic regression analysis (*p* < 0.05)

## Discussion

This study showed that patients who underwent simultaneous bilateral TKAs and patients with general anesthesia during TKA had a significantly higher risk of DVT after TKA even with LMWH use for chemoprophylaxis of DVT. Therefore, these patients should be carefully monitored for DVT after TKA.

It has long been widely debated whether prophylactic treatment for DVT is needed after TKA in Asians [4, 6, 19–21]. In earlier studies, the results were mostly against the use of prophylactic treatments unless symptomatic DVT developed [19]. However, more recently, studies showed that the DVT rate after TKA in the Korean population was increasing, with one study reporting a DVT incidence of up to 35.7% without prophylaxis [6, 22]. A recent Japanese study also reported a 62.5% DVT rate after TKA without prophylaxis [24]. Furthermore, some studies even recommended medical prophylaxis to Korean patients having risk factors such as obesity [2]. Because of the increasing incidence of DVT and the legal problems when PE, which could be fatal, occurs, today an increasing number of surgeons in Korea now use medical prophylaxis for DVT after TKA [6, 29]. We also acknowledge the risk of DVT and have used anticoagulant agents after TKA in our practice.

Several anticoagulant agents have been proposed for DVT prophylaxis after TKA. Today an increasing number of new agents have been invented and introduced to surgeons. Nonetheless, LMWH drugs such as enoxaparin and selective factor Xa inhibitors such as fondaparinux have been considered the standard therapy for prophylactic treatment for DVT [6, 8, 9, 25]. We have chosen and used one of these standard materials, enoxaparin, for DVT prophylaxis after TKA. The efficacy and safety of LMWH is well documented in the literature [30]. However, there is a lack of literature reporting the DVT rate after TKA with chemoprophylaxis in the Asian population. Chung et al. conducted a study with three different chemoprophylaxis regimens. The incidence rates of DVT were 7.3%, 15.5%, and 35.5%, respectively [6]. The DVT incidence rate of slightly more than 20% in our study is comparable with the results of Chung et al. and is lower than the results of previous studies without prophylaxis.

Risk factors for DVT after TKA have been studied and proposed by numerous investigators. Old age, female sex, obesity, and simultaneous bilateral cases of TKA are some of the well-known risk factors [2]. Rodgers et al. reported that epidural anesthesia reduced DVT incidence compared to general anesthesia [31]. Jiang et al. also reported that perioperative allogenic blood transfusion was significantly related to DVT following total joint arthroplasty [32]. However, there is a paucity of literature regarding DVT after TKA with the use of anticoagulation chemoprophylaxis in the Asian population. Because anticoagulant agents cannot prevent all cases of DVT after TKA, there should also be some risk factors for DVT even with prophylaxis. In this study, we found that general anesthesia and bilateral simultaneous cases were noticeable risk factors for DVT after TKA with chemoprophylaxis. Advanced age was not a significant factor, even in single-variable analyses. Although gender and BMI were notable factors in the single-variable analyses, they were not significant in the multiple logistic regression analyses.

There have been several studies comparing DVT incidence rates after TKA according to the anesthesia method. Most notably, Hu et al. reported in their meta-analysis study including 21 randomized controlled trials that epidural or spinal anesthesia significantly lowered the incidence of DVT when compared with general anesthesia. Their suggested reasons for this were altered coagulability, increased volume flow to the lower limbs, an improved ability to breathe without pain, and a reduction in surgical stress responses [33]. More recently, Pugely et al. reported no difference in DVT incidence rate between spinal anesthesia and general anesthesia. They used a large cohort database of more than 14,000 subjects: the American College of Surgeons National Surgical Quality Improvement Program database between 2005 and 2010 [34]. Ishii et al. also reported that no anesthesia method was better than any other in relation to the development of symptomatic DVTs and PEs in a Japanese population [35]. Our study result backs the one stating that, even with chemoprophylaxis, general anesthesia increases the risk of DVT after TKA.

It has long been considered that simultaneous bilateral TKA cases increase DVT incidence compared to staged bilateral TKA cases. A meta-analysis study by Fu et al. compared various clinical outcomes between simultaneous bilateral TKA cases with staged bilateral TKA cases, and one of the findings was that simultaneous bilateral TKA cases were related to higher thromboembolism rates. They cited longer use of pneumatic tourniquets, intramedullary guides, and cement as factors for high DVT rates [36]. Recently, Liu et al. reported in their meta-analysis study including 18 comparative studies that simultaneous bilateral TKA cases showed increased DVT incidence rates compared with staged TKAs [37]. Our study compared simultaneous bilateral TKA cases with only first leg staged bilateral cases or cases that planned to have just unilateral cases. We also found that simultaneous bilateral TKA increases the risk of DVT.

For the diagnosis of DVT, several modalities are available. Because the clinical diagnosis of DVT is somewhat difficult because of the vague symptoms, radiologic and laboratory tests are usually required. The serum D-dimer test has been considered as a useful screening test; however, because of its poor specificity, it has a limited value as a definite diagnostic method [2, 38]. Compression ultrasonography was reported to be safe and effective; however, there are concerns of accuracy issues [39]. Today CT angiography is widely considered a more sensitive tool in detecting DVT [40, 41]. We used CT angiography to secure an accurate diagnosis.

As for the timing of the DVT evaluation, the protocol in place for this study was the seventh postoperative day. There have been some variances among studies; however, most performed their DVT evaluation about seven days after the TKA. Kim et al. reported their results based on the sixth postoperative day DVT evaluations [2], and Park et al. reported the seventh postoperative day results [5]. Chung et al. stated that they performed the evaluation before the 10th postoperative day [6].

### Limitations

There are several limitations to this study. First, the risk factors for DVT that we used were selected rather arbitrarily. However, we referred to the literature for the selection of factors, and some of them were common choices for general clinical factor analysis studies. Second, a sample size of 103 was small for a factor analysis study. In retrospective observational studies like this one, a bigger sample size is always desirable. We hope to someday run a similar study with a much larger sample size. Third, because we did not perform lower extremity CT angiography before the operation, we could not differentiate whether the detected DVTs were old lesions or newly developed lesions after TKA. However, Chang et al. reported that preoperative DVT was rare and of limited clinical relevance [40]. Fourth, we did not investigate PE occurrence. If we had these data, it would have been a more complete study. However, because of the rareness of PE after TKA in Koreans [1, 14, 19], we suspected that it would be meaningless to investigate the PE occurrence with this sample size. Lastly, we did not collect data on patient symptoms related to DVT from their medical records. It would have been a much better and more complete study if we had presented data and analyses of DVT symptoms. Unfortunately, because this was a retrospective study, we could not collect reliable and constant data on symptoms in patients with DVT from the medical records.

## Conclusions

Patients who undergo simultaneous bilateral TKAs and patients who receive general anesthesia need more attention regarding DVT, even with DVT chemoprophylaxis using LMWH after TKA.

## Data Availability

The complete datasets used in this study are available from the corresponding author on adequate request.

## Abbreviations

DVT: deep vein thrombosis
TKA: total knee arthroplasty
LMWH: low-molecular-weight-heparin
ROM: range of motion
CT: computed tomography
BMI: body mass index
PT INR: prothrombin time/international normalized ratio
